# New views on phototransduction from atomic force microscopy and single molecule force spectroscopy on native rods

**DOI:** 10.1038/s41598-017-11912-8

**Published:** 2017-09-20

**Authors:** Sourav Maity, Nina Ilieva, Alessandro Laio, Vincent Torre, Monica Mazzolini

**Affiliations:** International School for Advanced Studies (SISSA-ISAS) via Bonomea 265, 34136 Trieste, Italy

## Abstract

By combining atomic force microscopy (AFM) imaging and single-molecule force spectroscopy (SMFS), we analyzed membrane proteins of the rod outer segments (OS). With this combined approach we were able to study the membrane proteins in their natural environment. In the plasma membrane we identified native cyclic nucleotide-gated (CNG) channels which are organized in single file strings. We also identified rhodopsin located both in the discs and in the plasma membrane. SMFS reveals strikingly different mechanical properties of rhodopsin unfolding in the two environments. Molecular dynamic simulations suggest that this difference is likely to be related to the higher hydrophobicity of the plasma membrane, due to the higher cholesterol concentration. This increases rhodopsin mechanical stability lowering the rate of transition towards its active form, hindering, in this manner, phototransduction.

## Introduction

Most available investigations on biological proteins are based on the use of Atomic Force Microscopy (AFM) and deal with purified proteins. In the present manuscript we analyze with AFM a natural membrane, in particular the membrane of the rod outer segments. The rod outer segment (OS) is a specialized biological structure - harboring the phototransduction machinery – which contains a stack of thousands of lipid discs surrounded by the plasma membrane^1^. How OS can perform phototransduction has been thoroughly investigated^2–7^ by using electrophysiology, biochemistry, and modeling. It is now clear that the OS is not homogeneous and that its properties are very different in its base and in its tip^8^. The plasma membrane and the discs have a different lipid and protein composition^9–15^. The concentration of cholesterol is higher in the plasma membrane^10^, determining its higher stiffness^16^ and a lower lateral diffusion^2,17,18^.

The composition of membrane proteins in discs and in the plasma membrane has been extensively studied^14^^.^ The most common membrane proteins in rod OS are CNG channels^17,19,20^ and rhodopsin, but the plasma membrane contains several other proteins, such as the Na^+^/Ca^2+^-K^+^ exchanger^19,21–23^, the calcium-activated TMEM16f lipid scramblase^24,25^, the glyceraldehyde 3-phosphate dehydrogenase, and Glut-1^23,24,26^. Other proteins, besides rhodopsin, are present in both the disc and the plasma membrane: the all-*trans*-retinol dehydrogenase, the GTPase- accelerating protein RGS9 and its membrane anchor R9AP, and the complex Rho-PDE^19,23,24,26^. Finally, some proteins localized at the rim region of the discs are rom-1, peripherin-2^19,22,23^, guanylate cyclase, and ABCA4^19,22,23^.

We here focus our investigation only on rhodopsin and CNG channels, by far the most common proteins in rod OSs. Native CNG channels^19–28^ are heterotetramers composed by three A1^29^ and one B1 subunits^30–32^ that share the same transmembrane topology. The C-terminal of both subunits harbors a cyclic nucleotide-binding (CNB) domain sharing 20% sequence identity with other cyclic nucleotide-binding proteins^33,34^. Rhodopsin^26,35^ belongs to the family of G protein-coupled receptors, with the C-terminal and N-terminal on the cytoplasmic and extracellular side of the membrane respectively. Its unfolding has been extensively investigated in discs isolated from bovine rod OS^6,36^. We have recently characterized the unfolding of heterologously expressed CNGA1 channels^7^. The unfolding of rhodopsin has been extensively investigated in discs isolated from bovine rod OSs demonstrating that the complete stretch (348 a.a.) from the N-terminal occurs at a tip- sample separation (TSS) of about 65 or 95 nm depending on the presence or the absence of a stabilizing disulphide bond between cysteine residues C110 and C187^6,36^. Rhodopsin molecules are present both in the discs and in the plasma membrane, but only rhodopsin in the discs is functional, i.e. initiates the phototransduction cascade^1,2^. Cyclic nucleotide-gated (CNG) channels are localized only in the plasma membrane^9,16^.

In this work, we use an approach that combines Atomic Force Microscopy (AFM) imaging, Single Molecule Force Spectroscopy (SMFS), and molecular simulations to explore the organization and the mechanical properties of the two most common membrane proteins of rod OSs: the CNG channel and rhodopsin. In the plasma membrane, we discovered highly organized structures formed by stable strings of tens of CNG channels. By using SMFS, we identified several rhodopsin molecules in the plasma membrane and in the discs, discovering that their Force-Distance (F-D or Force *versus* tip-sample separation) curves are strikingly different in the two environments. In order to elucidate the biochemical reason for this difference we performed molecular dynamics simulations of the unfolding of rhodopsin. These simulations suggest that the difference in the F-D curves can be ascribed to the higher cholesterol concentration in the plasma membrane. The higher cholesterol content increases hydrophobicity, enhancing the protein-lipids interactions, leading to a significantly higher stability of rhodopsin. We suggest that this is the key ingredient hindering the capability of rhodopsin to initiate phototransduction in the plasma membrane.

## Results

### Isolation and AFM imaging of rod OS plasma membrane and discs

Rod OSs were isolated from dark-adapted *Xenopus laevis* retinas; purified plasma membrane and discs were obtained (Figure SI 1a,b and Methods). By using low resolution AFM imaging (Figure SI 1b), we imaged the sample so to be able to localize and to identify the plasma membrane from the cytoplasmic side (Figure SI 1c,d,g,h), the discs formed by a single lipid bilayer (Figure SI 1e,i), named also open spread-flattened discs, and the intact discs formed by two lipid bilayers (Figure SI 1f,j).

**Figure 1:**
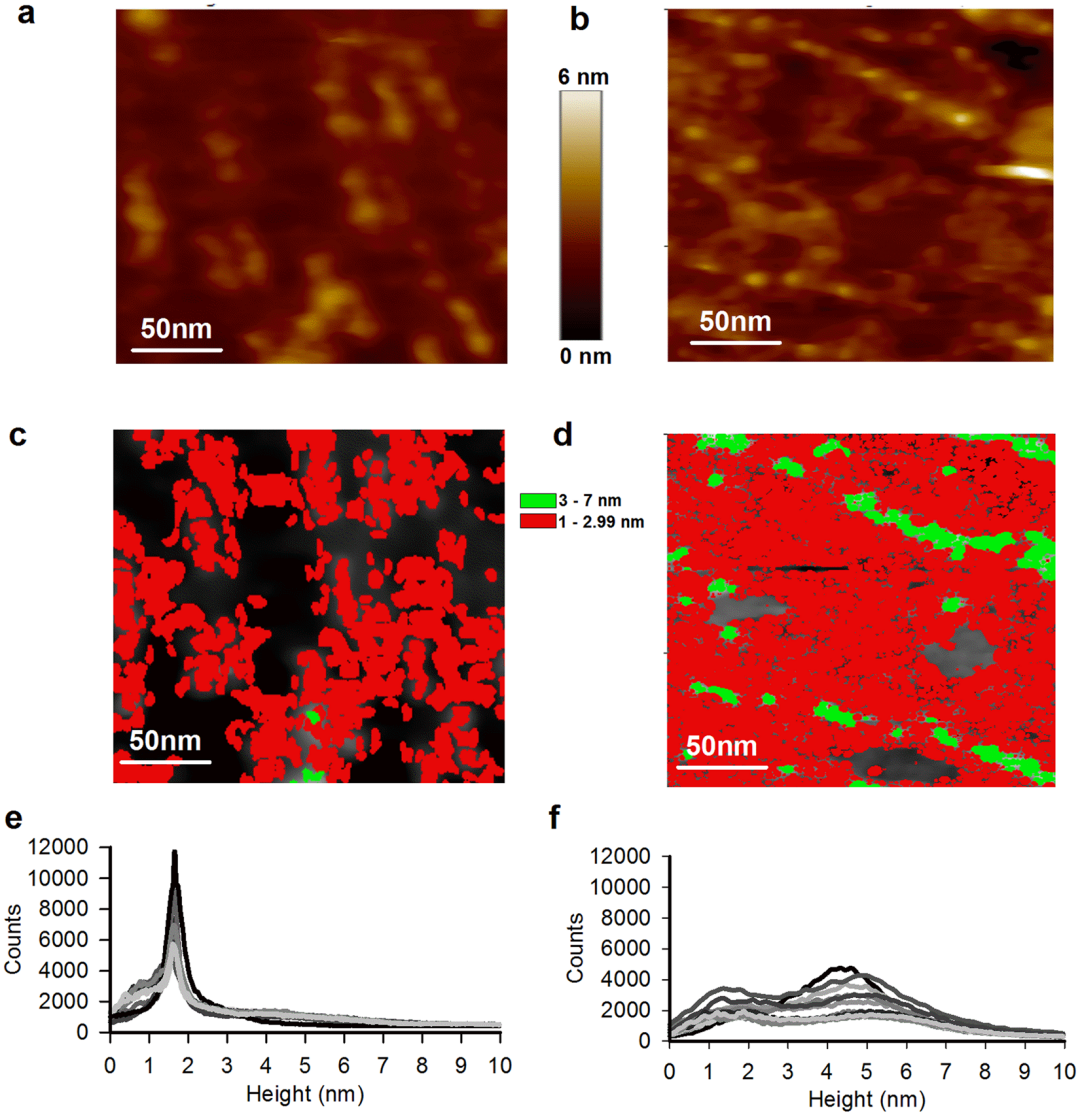
AFM image of membrane patches from the rod OS disc and the plasma membrane (**a**) AFM topography from rod OS disc membrane showing the organization of rhodopsin in the single layered disc membrane. (**b**) AFM topography from rod OS plasma membrane. (**c,d**) Clusterization of the images in panel a and b according to their height ranges from 1–2.9 nm (area with red color) and 3–7 nm (area with green color), respectively. (**e)** Superposition of histograms of height profile from all the detected particles as in panel c from the images taken from rod OS disc membrane in different experiments, with a mean protrusion of 1.5 ± 0.4 nm (n = 18). (**f**) Superposition of histograms of height profile from all the detected particles as in panel d, from the images taken from rod OS plasma membrane in different experiments, with two different protrusions: height profile of 1.5 ± 0.7 nm and 4.9 ± 0.9 (n = 22).

We imaged patches of 500 × 500 nm^2^ of the cytoplasmic side of the discs (Fig. 1a, open spread-flattened discs) and of the rod OS plasma membrane (Fig. 1b). Protrusions of the disc membranes had a height of about 1.5 nm (Fig. 1c) and those from the plasma membranes had a height between 1.5 to 4.9 nm (Fig. 1d) with a width of 5-10 nm. When these protrusions are colored according to their height, the spatial organization becomes evident: in both kinds of membranes there are patches with no significant protrusions (black in Fig. 1e,f), patches with a height between 1 and 2.9 nm (red), and the highest patches measuring between 3 and 7 nm (green). Protrusions with a height around 4.9 nm are often organized in strings made by a single file.

### Spatial organization of native CNG channels in the rod OS plasma membrane

**Figure 2:**
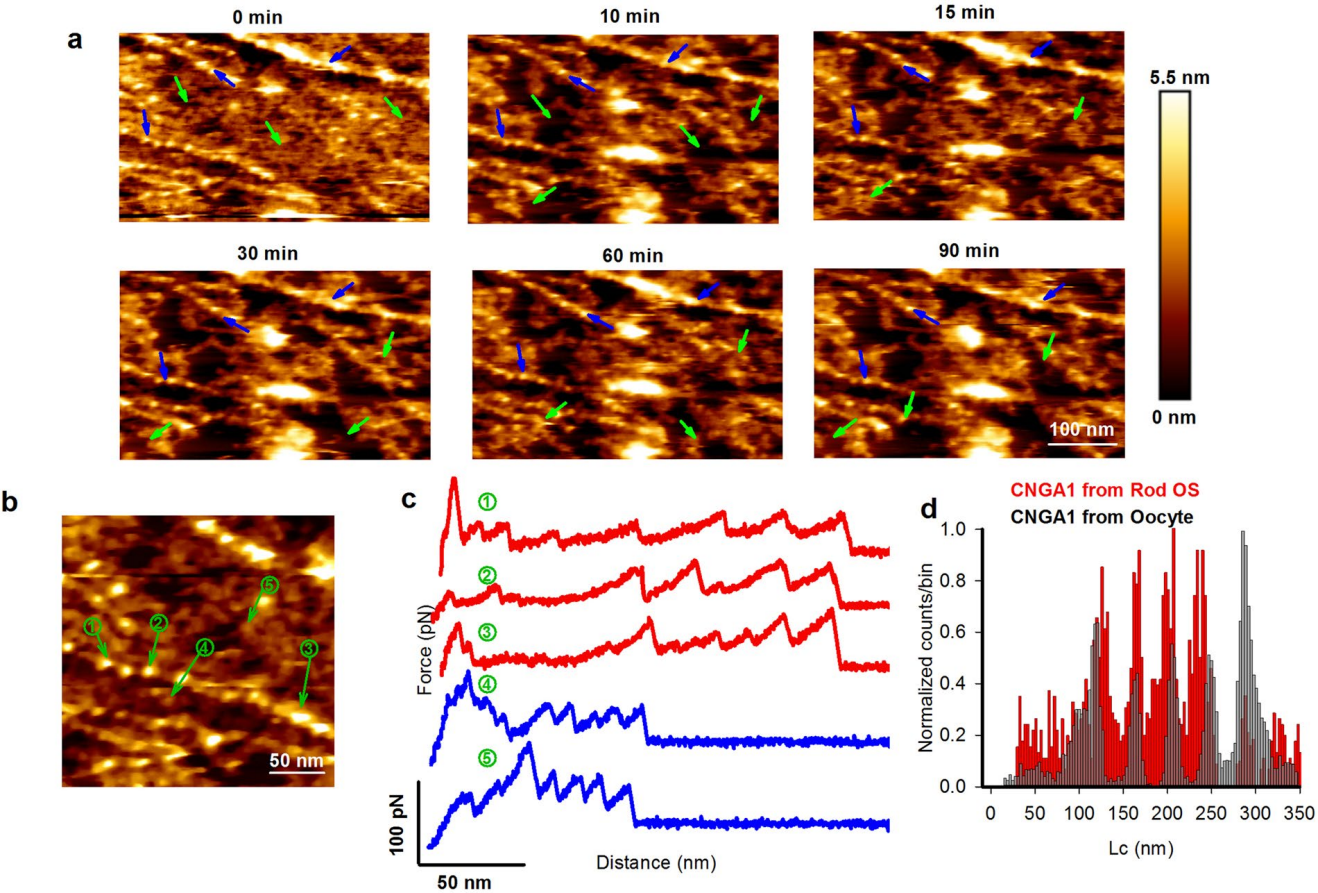
Identification of the spatial organization of CNG channels in the rod OS plasma membrane. (**a**) Selected membrane patch from the base of rod OS showing different topographical features. Acquisition of images at different times demonstrates stable (blue arrows) and unstable (green arrows) features. (**b**) High resolution image with protrusions from the lipid bilayer with different height; 1,2,3 are CNG channels while 4,5 are rhodopsin. (**c**) F-D curves obtained after pulling of selected protrusion (see numbered circles). (**d**) Superimposition of histograms of normalized counts/bin against Lc from 29 F-D curves (in red) of CNGA1 subunit from rod OS plasma membrane, and 157 F-D curves (in grey) of CNGA1 from oocyte plasma membrane.

Often, we obtain good AFM images (n = 5) from the same region at different times (Fig. 2a), so that we could identify which features were stable over time and whether some protrusions diffused. Membrane patches with no appreciable protrusions (see black regions), likely to contain no membrane proteins, were not stable and often disappeared (green arrows). High protrusions (white regions), in contrast, were usually organized in strings (blue arrows) and did not change their location over 1-2 hours, as if they were anchored to an underlying structure^22,37,38^ such as actin filaments^17,18^.

**Figure 3:**
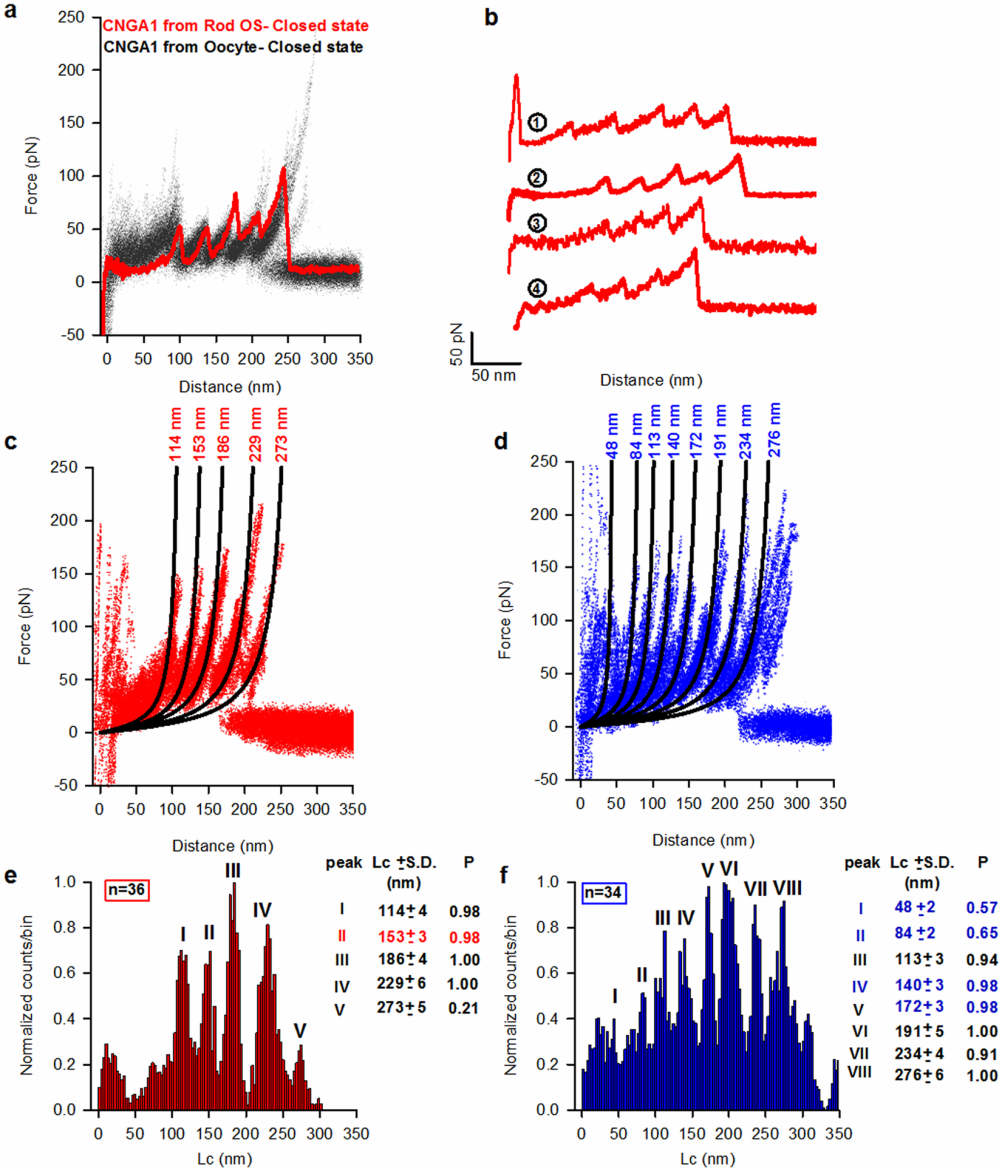
Single molecule unfolding of CNGA1 subunit in the closed and open states. (**a**) Superposition of 157 F-D curves (black lines) from oocyte plasma membrane overexpressing CNGA1 channels (modified from ref.^7^) and a single F-D curve of CNGA1 subunit (red trace) from rod OS plasma membrane, both in the absence of cGMP (closed state). (**b**) Representative F-D curves of CNGA1 subunit from rod OS plasma membrane; curves 1 and 2 are full length unfolding of CNGA1single subunit with an Lc value around 272 nm, while curves 3 and 4 are unfolding of a single CNGA1 subunit pulled up to S1 transmembrane domain with an Lc value around 231 nm. (**c**) Superposition of 36 F-D curves of CNGA1 subunit from rod OS plasma membrane in the absence of cGMP (closed state); continuous black lines are obtained from the fitting with WLC model and numbers indicate the corresponding values of Lc. (**d**) The same as in **c** but in the presence of 2 mM cGMP (open state). (**e**) Histogram of normalized counts/bin against Lc obtained from all the traces in panel **c** (bin-3 nm); numbers represent the corresponding Lc (mean±S.D, n = 36) values in nm and the relative probability (P): five force peaks with values of Lc equal to 114 ± 4, 153 ± 3, 186 ± 4, 229 ± 6, and 273 ± 5 nm; the frequency of occurrence is 0.98, 0.98, 1, 1.00, and 0.21, respectively, with an unfolding force of 95 ± 45 pN; number in red represents the characteristic peaks for CNGA1 channels in the closed state. In roman numerals the numbers of force peaks. (**f**) The same as in **e** but for all the traces in panel d; numbers in blue represent the characteristic peaks for CNGA1 channels in the open state: eight force peaks with Lc of 48 ± 2, 84 ± 2, 113 ± 3, 140 ± 3, 172 ± 3, 191 ± 5, 234 ± 4, and 276 ± 6 nm (n = 34), and higher unfolding force of 120 ± 39 pN (mean±S.D, n = 34); their frequency of occurrence is 0.57, 0.65, 0.94, 0.98, 0.98, 1.00, 0.91, 1.00, respectively.

In order to identify the molecular origin of these protrusions we performed SMFS experiments. Both kinds of protrusions appeared as blobs with the same width of about 8 nm but their height was either 4.9 ± 0.9 nm or 1.5 ± 0.7 nm (number of images n = 22; Fig. 2b, Fig. 1b), respectively. In some occasions, after AFM imaging, it was possible to perform SMFS with the same cantilever tip. We localized and unfolded both the high and low protrusions, which had a longer and a shorter unfolded length respectively (indicated by the numbered circles in Fig. 2b,c). This allowed to verify that F-D curves obtained from the unfolding of molecules from high protrusions and organized along strings (indicated by the numbered (1, 2, 3) circles in Fig. 2b,c) match very well the F-D curves obtained from the unfolding of homotetramer bovine CNGA1 channels - heterologously expressed in oocytes from *Xenopus laevis* ^7^. This allows to identify the high protrusions as native CNG channels with an intact cytoplasmic domain (see also Fig. 3). Some of the shorter F-D curves were later identified as the unfolding of rhodopsins. These results, obtained with AFM imaging and SMFS are in agreement with the observation – made with confocal microscopy - that native CNG channels in rod OS plasma membrane are not randomly distributed but are clustered in stable bundles with a width of some hundreds of nm. The strings of CNG channels seen in Fig. 2 could be guided by actin filaments^17,18^ to which CNG channels are anchored.

### Unfolding of CNG channels from the rod OS plasma membrane

We here discuss more in detail the unfolding properties of CNG channels in the Rod plasma membrane observed by SMFS. The bovine CNGA1 subunit has been investigated in detail by biochemical, electrophysiological and structural experiments^7,19–28,39^, and therefore, here, we will use it as a template. The bovine CNGA1 is composed of 690 a.a.^29^ while that from Xenopus rods has 708 a.a.^40^, and the two subunits have a high degree (86%) of sequence identity. F-D curves from rod OS plasma membranes were analysed and clusterized^7^. We considered the F-D curves with a Lc between 200-300 nm. Using the template from the unfolding of CNGA1 from oocyte^7^ (for example- gray curves with red curve in Fig. 3a) allowed the identification of 36 F-D curves representing the unfolding - from the C-terminal - of native CNG channels in the closed state (Fig. 3b,c,e) with a maximum of five force peaks, and 34 F-D curves representing the unfolding in the open state (Fig. 3d,f) with a maximum of eight force peaks.

In the closed state, the cytoplasmic domain of native CNG channels unfolds in a single step with an Lc value of 114 nm, while that of the CNGA1 subunit expressed in *Xenopus laevis* oocytes unfolds in more steps^7^. The force peak with a value of Lc at 153 nm corresponds to the unfolding of S6-P-helix-S5; the peaks with an Lc of about 186 and 229 nm correspond to the unfolding of S4-S3 and S2-S1, respectively. In the open state (Fig. 3d,f), the unfolding of native CNG channels is similar to the unfolding of the CNGA1 from the oocyte membrane^7^: the S6 helix unfolds in a single step at an Lc value of about 140 nm, then S5 and S4 unfold together at an Lc of about 172 nm^7^. The force peak with a value of Lc of about 191 nm corresponds to the unfolding of S3, followed by the complete unfolding of S2 and S1 at a value of Lc around 234 nm, and the detachment at 276 nm.

F-D curves obtained from the unfolding of native CNG and CNGA1 channels expressed in oocytes have force peaks with very similar values of Lc but that differ for two aspects. First, both in the open and closed states, the unfolding of CNG channels from rod OS plasma membrane occurs at higher forces (95 ± 45 and 120 ± 39 pN, respectively) than in oocytes (55 ± 20 and 80 ± 35 pN, respectively). Second, in the closed state, the detachment in approximately 90% of F-D curves from oocytes has an Lc value of about 276 nm^7^, corresponding to the total length of the subunit (690 a.a.). In contrast, in the closed state (Fig. 3c,e), the detachment in approximately 79% of F-D curves from rod OS has an Lc value of about 231 nm, corresponding to the unfolding of the last transmembrane segment S1 of the CNGA1 channel subunit. Therefore, membrane proteins expressed in a heterologous system have different properties to when they are embedded in their native environment.

### Identification and unfolding of rhodopsin from the rod OS plasma membrane and discs

**Figure 4:**
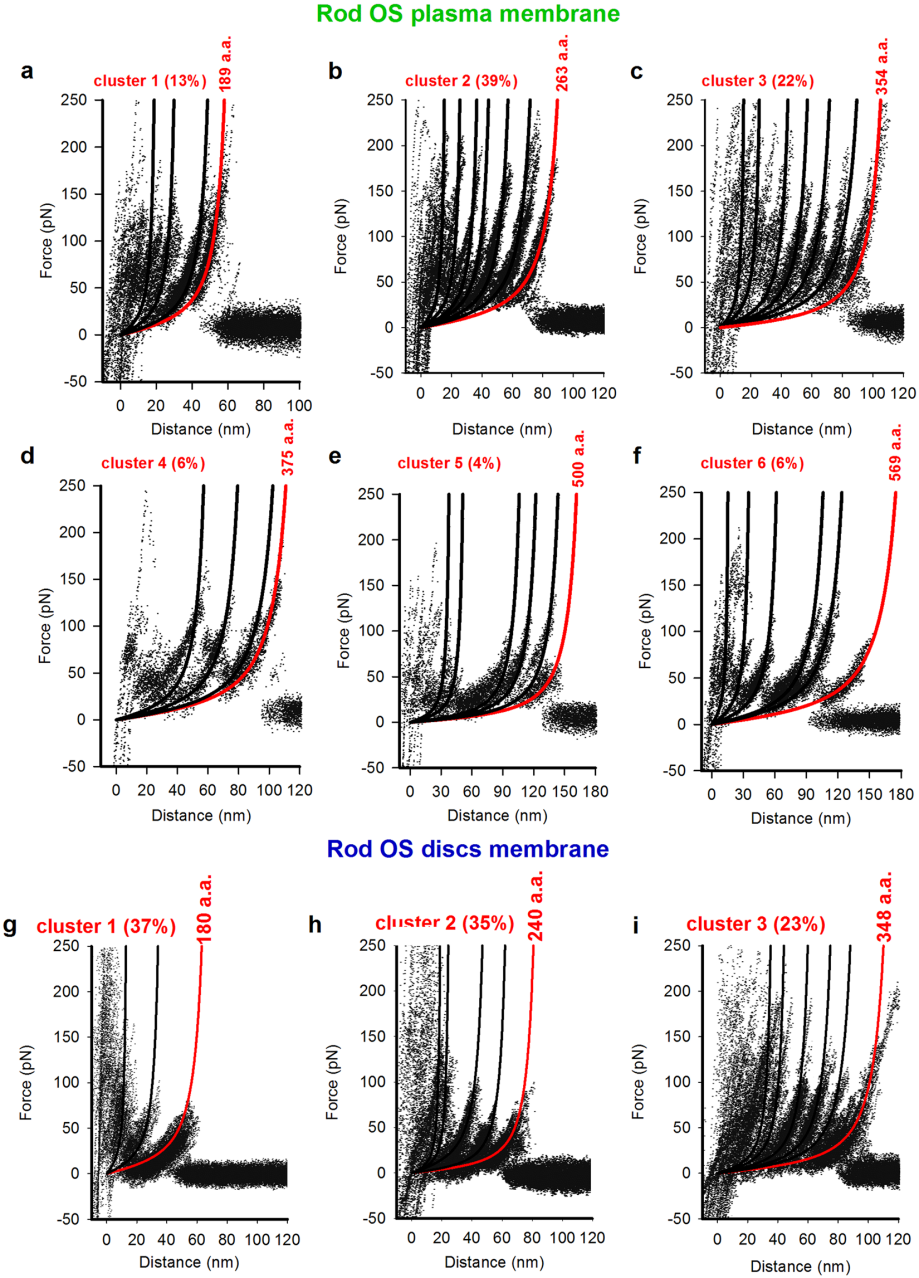
Clusters obtained from the SMFS experiment in the native rod OS plasma membrane and disc membrane at an Lc value lower than 200 nm. (**a**) Superposition of 40 F-D curves for Cluster 1 from rod OS plasma membrane; the continuous lines represent the fitting of WLC model to each force peak, where the red line represents the WLC fit to the final force peak, providing the complete length of the protein pulled by the AFM stylus; the a.a. number of residues corresponds to the last force peak position. The percentage represents the probability of occurrence (filtering procedure provided n = 311). (**b**–**f**) Superposition of F-D curves as in panel a but for the other Clusters; the percentage represents the probability of occurrence; filtering procedure provided n = 121, n = 68, n = 19, n = 16, n = 19, respectively. (**g**) Superposition of 92 F-D curves for Cluster 1 from rod OS disc membrane; the continuous lines represent the fitting of WLC model to each force peak, where the red line represents the WLC fit to the final force peak providing the complete length of the protein pulled by the AFM stylus; the a.a. number of residues corresponds to the last force peak position. The percentage represents the probability of occurrence (filtering procedure provided n = 249). (**h**,**i**) Superposition of 87 and 57 F-D curves, respectively, as in panel g but for the other Clusters.

Rhodopsin from *Xenopus laevis* retina is 354 a.a. long^41^, while that from bovine and mouse rods is 348 a.a. long^36,42,43^. To identify F-D curves obtained from the unfolding of rhodopsin from its C-terminal in rod OS plasma membrane, we filtered SMFS data taking into account two features: force peaks larger than 35 pN, and the last force peak with an Lc value not larger than 200 nm, corresponding to ~550 a.a. These F-D curves were clustered^7^ and we found 6 clusters with different total lengths (Fig. 4a–f) and occurrence frequency.

F-D curves of cluster 2 (n = 121) and cluster 3 (n = 68) had the highest occurrence, approximately 39% and 22% respectively. Their largest Lc value was about 85 and 127 nm, corresponding to the unfolding of a polypeptide chain of 238 and 354 a.a., respectively. Since the concentration of rhodopsin in the rod OSs is significantly higher than that of all other membrane proteins with a comparable number of a.a. – such as the enzyme glyceraldehyde 3-phosphate dehydrogenase (335 a.a. long) and Glut-1 composed of 12 TM domains (492 a.a. long), the all-*trans*-retinol dehydrogenase (312 a.a.), the GTPase- accelerating protein RGS9 (484 a.a.) – we identified F-D curves in cluster 2, as obtained from the unfolding of rhodopsin, where the Cys110-Cys187 disulfide bond was intact, and F-D curves in cluster 3 as traces where the Cys110-Cys187 bond was broken^36,42,44^. Following the same rationale, cluster 1 was postulated to represent a partial unfolding or rhodopsin while clusters 4, 5 and 6 presumably represented a partial or complete unfolding of unidentified membrane proteins present in the rod OS plasma membrane.

The C-terminal unfolding of rhodopsin from discs was analyzed with SMFS using intact discs (with a height of 14 ± 1.7 nm (n = 18) see also Fig. 1f) where the rhodopsin C-terminal was exposed both to the bathing medium and the cantilever tip. Analysis of SMFS data from intact discs allowed identifying three clusters (Fig. 4 g–i) ascribed to a partial unfolding of rhodopsin, with an Lc of about 65 nm (corresponding to 180 a.a. Fig. 4g cluster 1), to the unfolding of rhodopsin in the presence of the intact Cys110-Cys187 disulfide bond, with an Lc of about 86 nm (corresponding to 240 a.a. Fig. 4h cluster 2), and to the unfolding of rhodopsin in the absence of the Cys110-Cys187 disulfide bond, with an Lc of about 125 nm (corresponding to 348 a.a. Fig. 4i cluster 3).

**Figure 5:**
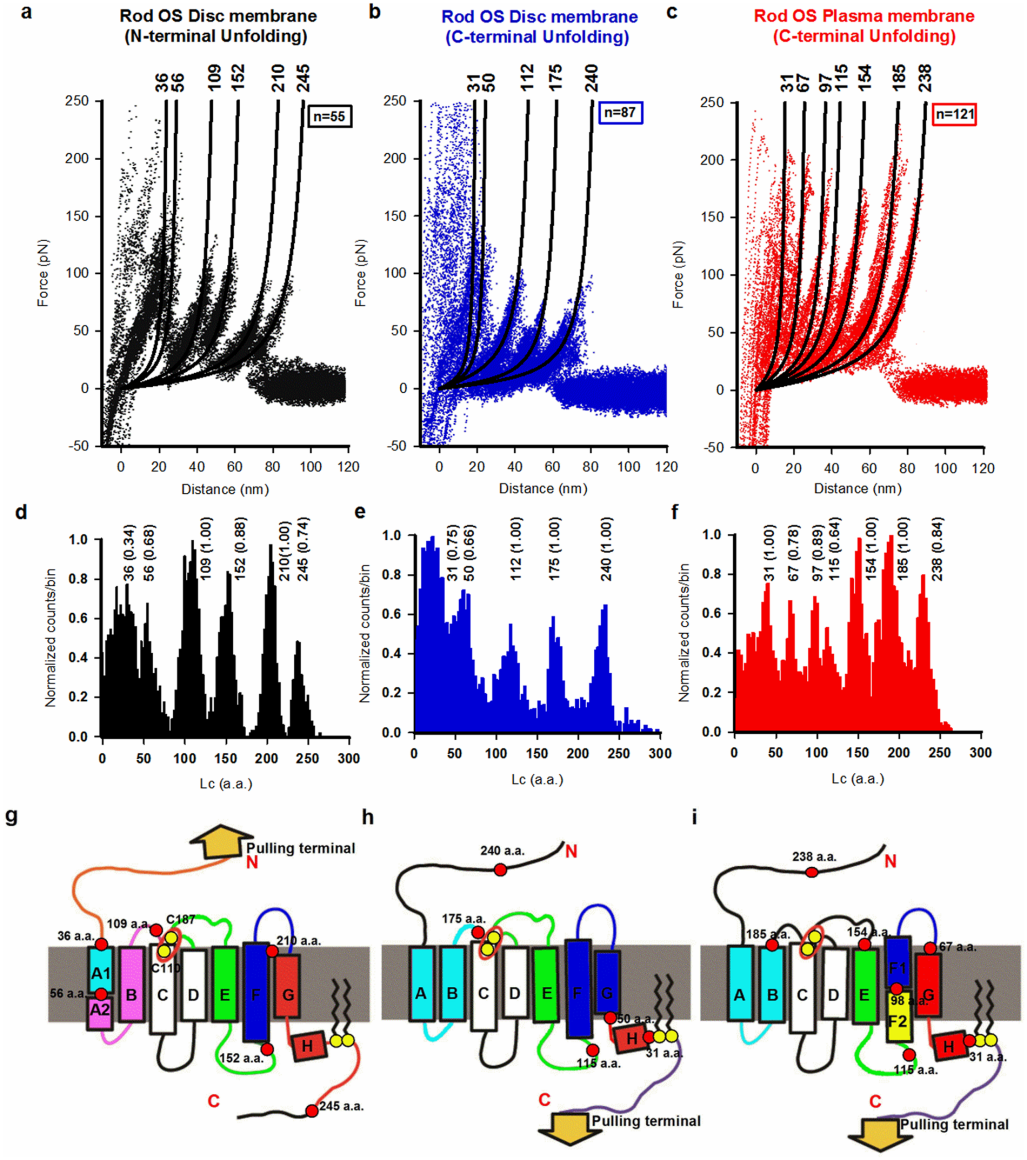
Unfolding pathways of native rhodopsin. (**a**) Superposition of 55 F-D curves obtained from the unfolding of a single rhodopsin from single layered open disc membranes (unfolding from N-terminal): six force peaks. The first force peak at Lc values of 36 ± 8 a.a. (n = 19), 56 ± 5 a.a. (n = 38), 109 ± 9 a.a. (n = 55), 152 ± 6 a.a. (n = 49), 210 ± 4 a.a. (n = 55), and 245 ± 10 a.a. (n = 40), with a probability of 0.34, 0.68, 1.00, 0.88,1.00 and 0.74, respectively, and the unfolding force of 130 ± 19 pN, 90 ± 26 pN, 80 ± 19 pN, 85 ± 22 pN, 62 ± 31 pN, and 65 ± 19 pN, respectively. Fit with the WLC model reveals the number of a.a. corresponding to force peak positions. (**b**) Superposition of 87 F-D curves as in panel a but from double layered intact disc membranes (unfolding from C-terminal) with five force peaks at Lc values of 31 ± 6 a.a. (n = 66), 50 ± 5 a.a. (n = 58), 112 ± 8 a.a. (n = 87), 175 ± 10 a.a. (n = 87), and 240 ± 8 a.a. (n = 87), with a probability of 0.75, 0.66, 1.00, 1.00, and 1.00, respectively. Force peaks are, respectively, 127 ± 19 pN, 98 ± 18 pN, 86 ± 15 pN, 55 ± 10 pN, and 57 ± 20 pN. (**c**) Superposition of 121 F-D curves obtained from the unfolding of a single rhodopsin from the cytoplasmic side (C-terminal) of the rod OS plasma membranes with seven force peaks at Lc values at 31 ± 6 a.a. (n = 121), 67 ± 4 a.a. (n = 95), 97 ± 4 a.a. (n = 108), 115 ± 8 a.a. (n = 78), 154 ± 8 a.a. (n = 121), 185 ± 10 a.a. (n = 121), and 238 ± 6 a.a. (n = 102), with probabilities of 1.00, 0.78, 0.89, 0.64, 1.00, 1.00, and 0.84 respectively. The unfolding forces are respectively: 118 ± 45 pN, 145 ± 30 pN, 145 ± 25 pN, 95 ± 20 pN, 150 ± 10 pN, 155 ± 15 pN, and 125 ± 30 pN. (**d**–**f**) Histogram of Lc values (in nm) of force peaks obtained from all curves in panel (**a**–**c**), respectively, (bin 0.8 nm or ~2 a.a.); numbers represent the Lc in a.a. and the frequency of occurrence for every peak. (**g–i**) Cartoon representation of the secondary structure of rhodopsin mapped with a structurally stable segment obtained with SMFS in panels a–c or in panels d–f respectively. Yellow circles represent native cysteins that are responsible for the structural stability of bovine rhodopsin. Red circles and corresponding numbers (in a.a.) represent the force peak positions of rhodopsin molecule unfolded from those three different situations.

The C-terminal unfolding of rhodopsin from discs and from the plasma membrane is remarkably different (Fig. 5). The unfolding in the open spread-flattened disc (Fig. SI 1e) and intact disc (Fig. SI 1f) has similar total Lc values and average unfolding forces (Fig. 5a,b,d,e), but they differ in their unfolding pathways^36^. On the other hand, the unfolding of rhodopsin from the plasma membrane requires comparatively larger forces, and the corresponding F-D curves have more force peaks (Fig. 5c,f). The unfolding from the plasma membrane has 7 major force peaks, whereas the unfolding from discs occurs with 5 major force peaks (Fig. 5b). The average force required to unfold rhodopsin from the plasma membrane and intact discs is 136 ± 135 and 74 ± 140 pN respectively (Fig. 5b,c). In contrast, the unfolding of rhodopsin from discs, either from the C-terminal or the N-terminal, requires similar forces: the average force required to unfold rhodopsin from its C- and N-terminal is 74 ± 14 and 76 ± 14.5 pN respectively (Fig. 5a,b). The number of major force peaks, however, is higher when rhodopsin is pulled from its N-terminal. The structural elements unfolded during each force peak are shown in Fig. 5g–i.

### Molecular dynamics simulations of the unfolding experiments

Differences of the unfolding of rhodopsin from discs and the plasma membrane are significant and require a molecular explanation. Rhodopsin in discs, when activated, initiates the phototransduction cascade, while rhodopsin in the plasma membrane does not^2,10,17,45^.

The most important difference between the two environments is the composition of the membrane, in particular its cholesterol content^9–12^. In order to elucidate the effect of this difference on the F-D curves, we performed molecular dynamics simulations of a model of the Rhodopsin-membrane system. We modeled rhodopsin^46,47^ by a coarse-grained sphere-like model using as input the coordinates of its native conformation in the PDB structure (1U19), and we simulated a pulling experiment by applying a constant velocity at the C-terminal, similarly to what had been previously done^48,49^. The same approach had also been used to model pulling experiments of Bacteriorhodopsin^49^ with a simplified model in which the membrane is kept rigid during the simulation and when the protein is pulled out of the membrane, it leaves a rigid hole that does not adapt to the new configuration, possibly altering the stability of the intermediate states of the unfolding. In order to circumvent this problem, we modeled the membrane with an extra potential energy term coupled to the distance z of the residues from the center of the membrane. This term favors the native value of z, mimicking in this manner the hydrophobic effect induced by the lipids (see Materials and Methods section). In our simulations, there is only one free parameter, i.e. the strength of the membrane potential ε_membr_: a larger value of ε_membr_ defines a highly hydrophobic membrane, such as a cholesterol-rich membrane.

**Figure 6:**
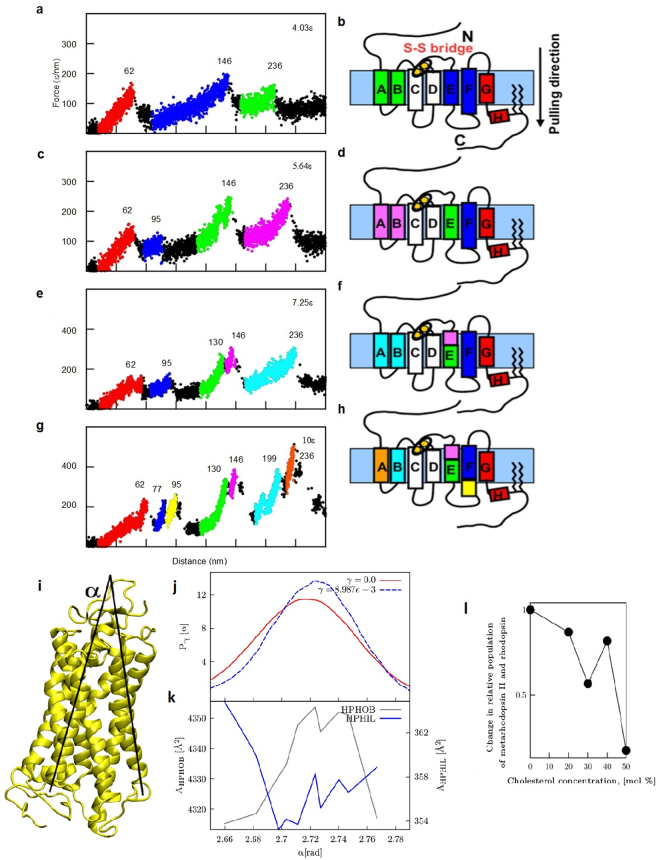
Simulation of unfolding pathways of rhodopsin and hydrophobicity of the membrane effects. **(a**,**c**,**e**,**g**) Simulated force-distance traces for bovine rhodopsin (PDB code 1U19) pulled by the C-terminal at k_B_T = 0.52 ε for different values of the parameter ε_MEMBR_ as indicated ((**a**) 4.03 ε, (**c**) 5.64 ε, (**e)** 7.25 ε, (**f**) 10 ε). Each plot represents the superposition of 10 traces obtained from 10 independent simulations. (**b**,**d**,**f**,**h**) cartoon representations of the order in which the transmembrane helices unfold in the simulations of the left panels, as derived by a visual inspection of the trajectories. The colour map is the same as for the traces. The numbers on top of each peak correspond to the length of the stretch that is unfolded up to the time step when the force drops (expressed in number of amino acids, n, see SI 4 for details). (**i)** The angle α between the transmembrane helices D and E. Panels j,k, and l show results theoretically derived from atomistic MD simulation of rhodopsin in DPPC bilayer without cholesterol. (**j**) Probability distribution of the angle α in a membrane without cholesterol (red line) and with 50% cholesterol concentration (blue dotted line); (**k**) Hydrophobic (A_HPHOB_), and hydrophilic (A_HPHIL_) transmembrane SASA of rhodopsin as a function of the angle α; (**l**) Change in the relative population of metarhodopsin II and rhodopsin $\frac{{P}^{METARHOD}(c)}{{P}^{RHOD}(c)}/\frac{{P}^{METARHOD}(0)}{{P}^{RHOD}(0)}$ as a function of the cholesterol concentration c in the lipid bilayer.

We performed four series of molecular dynamics (MD) simulations of unfolding of rhodopsin by varying ε_membr_ (Fig. 6a–h). By increasing the value of ε_membr_ from 4.03 ε to 10 ε the force required to unfold the protein becomes larger and larger, and the simulated F-D curves change their shape and more force peaks appear. For small values of ε_membr_ (4.03 ε), α-helices F, E, and B, A unfold together. For a higher value of ε_membr_ (5.64 ε), helices G, F, and E unfold sequentially, while A and B unfold together, exactly as when rhodopsin is pulled from the C-terminal in discs (Fig. 5). When the value of ε_membr_ is further increased (10 ε), all α-helices unfold sequentially – with the exception of D and C, which are linked together by a S-S bond^36^, and α-helices F and E that unfold in two steps, as experimentally observed when rhodopsin is unfolded from the plasma membrane (Fig. 5). The number of a.a. unfolded at each step obtained from the unfolding simulation is close to the number of a.a derived from the experimentally estimated value of Lc (Figure SI 4), both for SMFS experiments from discs (ε_membr_ = 5.64 ε), and from the plasma membrane (ε_membr_ = 10 ε). These results suggest that the differences of F-D curves when rhodopsin is unfolded from discs and from the plasma membrane can be ascribed to the more significant hydrophobicity of the plasma membrane, caused by its higher cholesterol content.

### Unfolding of rhodopsin from the OS plasma membrane treated with cyclodextrin

In order to confirm this scenario we performed SMFS experiments in the plasma membrane of rod OS treated with cyclodextrin, an agent known to chelate cholesterol^50,51^ and therefore to lower its concentration. The plasma membrane of rod OS was treated for 3 (n = 4) with 1 mM cyclodextrin and after washing out, SMFS were carried out for 2 hours (Figure SI 3). Following 3 minutes exposure to cyclodextrin, F-D curves corresponding to the unfolding of rhodopsin from its C-terminal were identified as previously described and we found that approximately 40% of these traces had 7 major force peaks and approximately 35% had 5 force peaks (Figure SI 3a–b). Therefore, the removal of cholesterol of the plasma membrane of rod OS shifts the number of force peaks – during the unfolding of rhodopsin – from 7 to 5 as usually observed when rhodopsin is unfolded from the discs (Figure SI 3e).

These results support the notion that the different number of force peaks during the unfolding of rhodopsin from discs and the plasma membrane can be ascribed to their different cholesterol concentration.

### The hydrophobicity of the membrane affects the conformational flexibility of rhodopsin and its activation

The transition of Rhodopsin to its active state, metarhodopsin II^52,53^ requires a series of conformational changes. The effect of membrane composition, and in particular cholesterol concentration, on similar conformational changes has been studied in detail, primarily for G-protein coupled receptors^54,55^. By using MD simulations we argue that cholesterol also alters the flexibility of rhodopsin and the related equilibrium between its active and inactive forms. If the membrane is more hydrophobic, the configuration with more exposed hydrophobic residues will be favored reducing also the extent of conformational fluctuations and the ability of Rhodopsin to become active. To test this hypothesis, we performed all-atom MD simulations of the inactive conformation of rhodopsin in dipalmitoylphosphatidylcholine (DPPC) bilayer for 70 ns at a temperature of 323 K (see Supplementary info 5) and we measured the angle α between the transmembrane helices D and E (see Fig. 6i), thought to be a key descriptor for the G-protein activation^56^. We also estimated the solvent accessible surface area (SASA) of the hydrophobic transmembrane residues, *A*_*HPHOB*_, and the SASA of the hydrophilic transmembrane residues, *A*_*HPHIL*_, using the program Free SASA^57^. The probability distribution of α (see red line in Fig. 6j) is characterized by a broad maximum at about 2.72 rad. Remarkably, *A*_*HPHOB*_ and *A*_*HPHIL*_ have a maximum and a minimum – respectively - approximately in correspondence with the most probable value of α (see Fig. 6k). This implies that in the most likely conformations rhodopsin exposes to the membrane the largest number of hydrophobic residues allowed by its fold and the minimum possible number of hydrophilic residues. A membrane with a higher hydrophobicity will hamper large fluctuations, therefore favoring the native conformation. To quantify this effect, we exploited the estimate from ref.^58^ of the partition coefficient of a triethylamine, a hydrophobic molecule, from a pure membrane to a membrane containing various concentrations of cholesterol. Using this estimate, we determined the change in the probability of different SASA configurations as a function of the cholesterol concentration and the associated distribution of the values of α induced by cholesterol in the membrane (see Figure SI 5). High cholesterol content shifts the distribution to the right and makes it slightly narrower, favoring the conformations with more hydrophobic residues exposed towards the membrane, and hindering fluctuations of α. We noted that larger distortions of the probability distribution would imply a change in the tertiary packing of the helices. This would be inconsistent with the experimental observations, and indeed we did not observe it in our simulations. A change of approximately 0.18 rad in α corresponds to a change of approximately 9.5 Å in the distance between C_α_ 159 and 222. Therefore, a tiny change in the distribution of the angle α, leads to a significant difference in the structural ensemble.

Due to the hydrophobicity effect, the cholesterol concentration changes the relative ratio between metarhodopsin II and rhodopsin (Fig. 6l) and therefore the rate of rhodopsin activation. At a concentration of 50% the ratio between metarhodopsin II and rhodopsin is approximately 0.17 of the ratio observed in the absence of cholesterol, but even at a lower concentration the ratio of the two populations is affected.

## Discussion

In the present work we illustrate how AFM imaging and SMFS can be combined to reveal new properties of proteins embedded in natural membranes, in particular those found in the outer segments of rod photoreceptors. The investigation was carried out on a sample which was neither purified nor crystallized. This shows that AFM imaging and SMFS can be used also on native membranes^36^ which are heterogeneously populated. Rod outer segments are highly specialized biological structures with a limited number of different proteins, so that we could identify CNG channels and rhodopsin both in the plasma membrane and in the discs.

We discovered that native CNG channels are not randomly distributed but form strings of single file molecules. Confocal microscopy has shown that native CNG channels localised in the rod OS^17,18^ are anchored to actin filaments, so that CNG channels remain confined in the plasma membrane and are therefore excluded from the discs. This compartmentalization is an essential component of the morphogenic programs operating in the rod OS^17,18^. These actin filaments have a width of some hundreds of nanometers, significantly larger than the single file of CNG channels seen with AFM (Fig. 2). Therefore, our results suggest that thinner actin filaments may branch from those seen with confocal microscopy and form a network in the plasma membrane where CNG channels are anchored. If CNG channels were present in the discs, their modulation by cyclic nucleotides and their consequent opening and closing would not contribute to the generation of electrical signals: indeed, only the opening and closing of ion channels in the plasma membrane influence the membrane potential of the rod photoreceptors, which controls the electrical signaling to second order neurons. The anchoring of CNG channels is also beneficial to the efficacy of phototransduction: indeed, a strong Brownian diffusion of CNG channels increases the variability in the number of CNG channels closed by a single photo-isomerization, and therefore their anchoring to the OS plasma membrane will make the single photon response more reproducible. As suggested elsewhere, such anchoring might provide a platform coordinating the spatio-temporal interaction of signaling molecules^59^.

SMFS allows to identify the protrusions observed by AFM and, even more importantly, to study the mechanical properties of the membrane proteins in a native-like environment. The unfolding of native CNG channels from OSs is similar – but not identical – to the unfolding of the bovine CNGA1 subunits expressed in *Xenopus laevis* oocytes.

Native CNG channels are composed of the assembly of three CNGA1 subunits and one CNGB1 subunit^19,20^. Native CNG channels from *Xenopus laevis* and homotetrameric bovine CNGA1 channels - expressed in oocytes^7^ - have the same kind of unfolding but differ in their detachment: F-D curves obtained from the unfolding of CNGA1 subunits have force peaks with values of Lc very similar to those obtained from the unfolding of native CNGA1 subunits but with higher values of force peaks. CNGA1 and CNGB1 subunits differ primarily at the N-terminal, where the CNGB1 has the large cytoplasmic domain GARP^20^, but have a very similar organization of the transmembrane domain and of the cytoplasmic C-terminal region. The unfolding from the C-terminal of the CNGA1 and CNGB1 subunits from native CNG channels is likely to be similar and it is possible that some of the F-D curves (Fig. 3) were obtained from CNGB1 subunits.

The unfolding of native CNG channels and of the CNGA1 subunits expressed in oocytes have a consistent difference in the detachment: in the closed state, in native CNG channels detachment occurred at a value of Lc of about 229 nm, corresponding to the unfolding of S1, but in CNGA1 channels expressed in oocytes the detachment normally occurred at an Lc value of about 276 nm^7^ – corresponding to the total length of the subunit (690 a.a.) - and only in about 10% of F-D curves detachment occurred at an Lc of 234 nm. Therefore, the N-terminal of native CNG channels is likely to be free more often than that of CNGA1 subunits expressed in oocytes. The unfolding of CNGA1 channels from the plasma membrane of rod OSs requires larger forces than those necessary to unfold CNGA1 channels expressed in oocytes (compare Fig. 2 with ref.^7^), presumably due to the anchoring effect of the actin network present in rod OSs.

We also find that the unfolding pattern and the mechanical stability of rhodopsin from discs^60^ and OSs plasma membrane is remarkably different: larger forces are needed to unfold rhodopsin from the plasma membrane and more force peaks are present in the F-D curves.

Our experiments (Fig. 5 and Fig. SI 3) and simulations (Fig. 6) contribute to answering the long-standing question: why doesn’t rhodopsin in the plasma membrane initiate phototransduction?^2,59^

MD simulations of the unfolding of rhodopsin – described as a suitable chain of identical elements (Fig. 6a–h) – show that the unfolding from the plasma membrane requires larger forces because of the stronger interactions with the lipid environment: these stronger interactions determine an increased mechanical stability^61^, altering rhodopsin’s flexibility and its ability to change conformation. We performed all-atom MD simulations of rhodopsin embedded in a DPPC lipid bilayer. These simulations suggest that the higher cholesterol content might shift the equilibrium ratio between rhodopsin and its active form metarhodopsin towards the inactive state. Therefore, we conclude that the higher content of cholesterol in the plasma membrane is the cause for the local rhodopsin’s inability to initiate the phototransduction cascade.

Rod photoreceptors have an exquisite sensitivity and are among the most efficient detectors. The present investigation reveals some of their underlying molecular mechanisms: i – CNG channels are confined in the plasma membrane where their modulation determines phototransduction; the opening and closing of CNG channels in the discs is wasteful because their modulation will not contribute to the membrane voltage of rod photoreceptors; ii – rhodopsin, which is localized on both membranes, is able to perform its function only in the membrane with a low cholesterol concentration where it can diffuse efficiently contributing to the high gain of the phototransduction cascade. Cone photoreceptors, in which rhodopsin and CNG channels are localized in the same membrane, rich in cholesterol, have a much lower sensitivity. Therefore, the molecular mechanisms described in this manuscript provide new clues to understand how rods achieve their high sensitivity and clarify the functional differences between rods and cones.

The present investigation, carried out using the membranes of rod OSs, can be repeated in other neuronal native membranes to reveal, with molecular resolution, the architecture and dynamics of membrane proteins in their natural environment.

## Materials and Methods

### Experimental setup

The AFM (JPK NanoWizard 3) was mounted on an Olympus IX71 inverted microscope^7^. The stage of the microscope contained a liquid chamber which was connected through the inlet-outlet channels to a liquid pump. The pump had a range of operation of 0.02 to 3 ml/min. The illumination for the microscope was done under dim red light with a wavelength of 820 nm. The setup was mounted over an anti-vibration table and was covered by an acoustic hood, useful to isolate and maintain dark conditions and to increase the stability of the system.

### Mechanical dissociation of photoreceptors

Dissociated rod photoreceptors (Fig. SI 1a) were obtained from adult male *Xenopus laevis* (“Xenopus express” Ancienne Ecole de Vernassal, Le Bourg 43270, Vernassal, Haute Loire, France) as previously reported^8^. Sample preparation and AFM experiments were done in the presence of infrared illumination (wavelength: 820 nm). Two to three frogs were dark-adapted overnight, and after anaesthesia with MS-222 (tricaine methanesulfonate) the eyes were surgically extracted. Eyes were preserved in Ringer solution containing (in mM): 110 NaCl, 2.5 KCl, 1 CaCl_2_, 1.6 MgCl_2_, 3 Hepes-NaOH, 0.01 EDTA, and 10 glucose, (pH 7.8 buffered with NaOH). The eye was then enucleated and hemisected under a dissecting microscope and the extracted retina, isolated from the pigment epithelium, was maintained in the Ringer solution. A small piece of retina was then transferred to an absorption buffer containing (in mM): 150 KCl, 25 MgCl_2_, and 10 mM Trizma base (pH 7.5). Isolated and intact rods were obtained by mechanical dissociation and were deposited on freshly cleaved muscovite mica, placed in the liquid chamber mounted on the AFM stage. All experiments performed in this study were approved by the International School for Advanced Studies Ethics Committee according to the Italian and European guidelines for animal care (D.L.26, March 4th 2014 related to 2010/63/UE).

### Preparation of rod plasma membrane

Incubated rods were maintained for 30–45 minutes over the mica in order to be adsorbed by its negatively charged surface. The surface adhesion increases in the presence of high concentration of Mg^2+^ ions in the absorption buffer (in mM): 150 KCl, 25 MgCl_2_, 10 Trizma base, pH 7.8. The surface was monitored by means of the inverted microscope. Subsequently, the surface was cleaned properly with an absorption buffer for 2 minutes at a flow rate of 0.5 ml/min. The surface was then monitored again. In this step, all the rod cells that were loosely bound to the surface were removed, leaving only the cells strongly attached to the surface. The AFM head was mounted placing the tip over a healthy rod outer segment (OS), far from the surface (~500 μm). After the selection of a good cell, an enzyme mixture of 100 μM Hyaluronidase and 1 μM/ml Neuroaminidase was injected into the chamber for 5 minutes using the pump. After the enzymatic treatment, the surface was cleaned immediately with a solution containing (in mM): 150 KCl, 10 Tris-HCl, (pH 7.5) at a liquid flow rate of 1-2 ml/min. Using this procedure (usually after 5-8 minutes) the cytoplasm of the attached rod was removed leaving only the plasma membrane attached to the mica surface. The surface was then cleaned gently (flow rate of 0.06 ml/min), first with the absorption buffer, to remove debris which could have gathered on top, and then with the recording solution. The obtained patches of rod OS plasma membrane were then ready for AFM measurements. Each day an experiment was performed, freshly extracted eyes were used and the reported results for Rod-OS plasma membrane were acquired from at least 20 different days of experiments. All the AFM experiments (imaging and SMFS) were carried out at a temperature maintained at 25 °C using BioCell^TM^ (temperature controller) from JPK Instruments.

### Imaging and identification of rod OS plasma membrane

After sample preparation, the AFM tip (HYDRA 50NGG from App-Nano, spring constant ~0.08 N/m) was brought back to the surface. AFM images were taken using tapping mode at an operating frequency of ~14 kHz, in the presence of the recording solution, on a surface area of 50 μm^2^. About 80% of the attempts successfully localized a long patch of protrusion with a height profile of ~8 nm and a diameter of ~5 μm considered to be a rod OS plasma membrane (Figure SI 1b–d). A further higher resolution image was taken on top of the membrane in an area of 1-2 μm^2^ (Figure SI 1d). Then the same surface area was taken for the SMFS experiments.

### SMFS on rod OS plasma membrane

SMFS was performed using the same tip as for imaging; the cantilever was calibrated by thermal fluctuation method^62^ before each experiment. Each approach of the cantilever was maintained at a constant speed of 1 μm/s, then the tip was pushed to the surface with a force of 1 nN for 0.5 s and retracted with a constant speed of 500 nm/s up to a distance of 500 nm. This process of approach and retraction continued for several thousands of times for different points in each scanning area, while the deflection of the cantilever was recorded as a force *versus* distance (F-D) curve (-or Force *versus* tip-sample separation). F-D curves were saved automatically. After acquisition of ~200.000 F-D curves, we performed the bio-informatics analysis^7^. The clusterization provided possible clusters of F-D curves coming from the unfolding of the variety of proteins that are present in rod OS plasma membrane. Initially, we converted each F-D curve to an F-Lc histogram by solving point by point the WLC equation with a persistence length of 0.4 nm as in Ref 0.^7^. These histograms were then processed to form clusters as described in ref.^7^. We considered that the variability of the non-specific tip-sample attachment was within 5 nm and therefore F-Lc histograms and F-d curves could be shifted by no more than 5 nm so to obtain an optimal similarity/superposition. More details on the used procedures are described in ref.^7^. Each cluster was then separated according to the unfolded length (in nm). Different clusters were evaluated separately. For extraction of molecular information from the F-D curves we used the contour length histogram (Lc). In order to obtain the number of single a.a. unfolded at a force peak with a given value of Lc, we divided the value of Lc by 0.4 nm, considered to be the average length of a single a residue in a stretched peptide chain. The sequence identity - obtained with BLAST (https://blast.ncbi.nlm.nih.gov/Blast.cgi) - between the CNGA1 subunit cloned from bos taurus^29^ (690 a.a. long) and the CNGA1 subunit predicted from *Xenopus laevis* ^40^ (708 a.a. long) is 86%. Data are usually given as the mean ± S.D. and represent the Lc (in nm) in order to have easy comparison with the already published analysis^7^. The probability was calculated using the Procedure explained in Fig. SI 2.

### Isolation, imaging and SMFS of disc membranes

Dark-adapted adult male *Xenopus laevis* frogs were used for the isolation of disc membranes. Modifying a previously reported protocol^63^, 8 retinas were isolated, and discs were separated with a first centrifugation (centrifuge Avanti J-26 XP Beckman Coulter with the Swinging-bucket Rotor JS-24.15) of the sample overlaid on a 15-40% continuous gradient of OptiPrep (Nycomed, Oslo, Norway). The harvested band was then collected and a second centrifugation with a gradient of 15-40% was done to obtain the purified membrane (Fig. SI 1e,f). For AFM imaging and force spectroscopy 20 µl of the sample were diluted with 20 µl of absorption buffer and were incubated on freshly cleaved mica for 30 minutes. After incubation, the surface was gently cleaned with recording solution containing (in mM): 150 KCl, 10 Tris-HCl, (pH 7.5). The AFM imaging, SMFS, and data acquisition were done following the same procedure described above. Data are usually given as the mean ± S.D and represent the a.a in order to have easy comparison with the already published analysis^36,42,44^. The probability was calculated using the Procedure explained in Fig. 2- figure supplementary 2.

Each day an experiment was performed it was done with freshly extracted eyes and the reported results for Rod-OS plasma membrane were acquired from at least 20 experiments performed in different days. All the AFM experiments (imaging and SMFS) were carried out at a temperature maintained at 25 °C using BioCell^TM^ (temperature controller) from JPK Instruments. The reported set of data for Rod disc membrane were acquired from at least 15 experiments in different days from 5 different sets of isolated disc preparations.

### Coarse-Grained Molecular Dynamics Simulations

The potential energy of the coarse-grained system used to simulate the unfolding experiments is given by

1 $${E}_{p}(x)={V}^{BB}+{V}^{NAT}+{V}^{NON}+{V}^{CHIR}+{V}^{MEMBR}$$

The amino acids in the protein are represented as single beads centred in the positions of the C^α^ atoms. The backbone potential acting on two consecutive beads is harmonic and tethers them at the peptide bond length, d_0_ = 3.8 Å.

2 $${V}^{BB}=\sum _{i=1}^{N-1}{k}_{BB}{({r}_{i,i+1}-{d}_{0})}^{2}\,$$

Where ${r}_{i,i+1}={r}_{i}-{r}_{i+1}$ is the distance between two adjacent beads and *k*_*BB*_ = 33.34 ε/Å^2^ is the spring constant. This is the smallest value of *k*_*BB*_ that allows us to use a large timestep of 15 fs. In ref.^47^,the authors used k_BB = 100 but with this value in our simulations the system becomes unstable.,

The V^NAT^ and V^NON^ terms take into account the non-bonded interactions. In order to distinguish between native and non-native interactions we proceed as follows. We assume that a pair of atoms occupies a spherical space corresponding to the sum of the vdw radii of the atoms enlarged by the factor 1.244 (ref.^64^). If the native separation between these atoms is smaller than the sphere’s radius a native contact is assumed to be present. This check is performed on every heavy atom in the PDB structure. The interaction between residues that form a native contact is described by the Lenard-Jones potential:

3 $${V}^{NAT}=\sum _{i < j}^{NAT}4\varepsilon [{(\frac{{\sigma }_{ij}}{{r}_{ij}})}^{12}-{(\frac{{\sigma }_{ij}}{{r}_{ij}})}^{6}]$$

Here, r_ij_ is the distance between the C^α^-s of the residues and the sigma parameters correspond to their distance in the native structure. The parameter, ε, fixes the scale of the energy, and is assumed to be equal to 1. The non-native interactions are assumed to be purely repulsive for distances shorter than d_cut_ = 5.61 Å with σ_0_ = 5 Å:

4 $${V}^{NON}=\sum _{i < j}^{NON}{V}_{ij}$$

5 $${V}_{ij}^{NON}=4\varepsilon [{(\frac{{\sigma }_{0}}{{r}_{ij}})}^{12}-{(\frac{{\sigma }_{0}}{{r}_{ij}})}^{6}]+\varepsilon ,{r}_{ij} < {d}_{cut}$$

6 $${V}_{ij}^{NON}=0,\,{r}_{ij} > {d}_{cut}$$

To account for the native chirality we also include a term of the form

7 $${V}^{CHIR}=\sum _{i=2}^{N-2}{k}_{CHIR}{C}_{i}^{2}\Theta (-{C}_{i}{C}_{i}^{NAT})$$

where Θ is the step function (1 for positive arguments and −1 otherwise), the chirality of the residue i is given by

8 $${C}_{i}=(\frac{({v}_{i-1}\times {v}_{i})\cdot {v}_{i+1}}{{d}_{0}^{3}}),{v}_{i}={r}_{i+1}-{r}_{i}$$

and C_i_^NAT^ is the chirality of residue i in the native structure. *k*_*CHIR*_ is equal to ε in agreement with ref.^47^.

To model the effect of the membrane, we introduce an additional term in the potential energy V^MEMBR^ applied only along the z-axis and which acts in a different manner on the residues based on their chemical nature and on their native contacts with the membrane. By using the Kyte and Doolittle hydrophobicity scale we divide the residues into hydrophobic and hydrophilic, and based on the experimental structure we determine which are in contact with the membrane. Our membrane potential is acting on the hydrophobic residues in contact with the membrane in the following way:

9 $${V}^{MEMBR}=\{\begin{array}{c}{\varepsilon }_{MEMBR(\frac{{z}_{min}-{z}_{i}}{3})for({z}_{min}-3{\rm{\AA }}) < {z}_{i} < {z}_{min}}\\ {\varepsilon }_{MEMBR(\frac{{z}_{i}-{z}_{max}}{3})for{z}_{max} < {z}_{i} < ({z}_{max}+3{\rm{\AA }})}\\ 0\,for\,{z}_{min} < {z}_{i} < {z}_{max}\\ {\varepsilon }_{MEMBR}\,otherwise\end{array}$$

The borders of the membrane are denoted as z_min_ and z_max_. We here take z_min_ = −17.034 Å and z_max_=15.896 Å. The same potential multiplied by −1 acts on hydrophilic residues. For details on the validation of the functional form of V^MEMBR^ see SI 5.

Our system evolved in time through the Langevin equation:

10 $$m\ddot{r}=-\gamma \dot{r}+{F}_{c}+\sqrt{2\gamma {k}_{B}T}\xi $$

Such equation was applied to each bead along the chain. We took m=1 and γ = 8.14.10^−4^ fs^−1^. F_c_ was the net force due to molecular potentials and external forces and $\xi$ was a Gaussian noise term. These equations of motion were solved with the Velocity Verlet integration scheme with timestep dt = 15 fs. In order to fix the effective temperature of our system, we performed several simulations of our model of rhodopsin at different temperatures. In this manner we discovered that the unfolding temperature is 0.65 ε. Knowing that usually the melting temperatures of membrane proteins is 10–20% above room temperature, we took k_B_T = 0.52 ε.

For the pulling simulations, we introduced an additional harmonic spring attached to the C-terminal of rhodopsin, while the N-terminal was kept fixed. The outer end of the spring was pulled at constant velocity v_pull_ = 2.035.10^−6^ Å/fs. The separation of the moving end from its origin corresponded to the cantilever displacement in the AFM experiment.

### Ethical Approval

All studies were approved by the SISSA’s Ethics Committee according to the Italian and European guidelines for animal care (D.L.26, March 4th 2014 related to 2010/63/UE). Concerning the dissociated rod photoreceptors, cells were obtained from an adult male Xenopus laevis (“Xenopus express” Ancienne Ecole de Vernassal, Le Bourg 43270, Vernassal, Haute Loire, France). The frogs were dark-adapted and the eyes were enucleated and emisected. Before this surgical operation the animal was killed through anesthesia overdose via 0.5 h of immersion in a 5 g/l MS-222 solution adjusted to pH 7.4. Oocytes were harvested from female Xenopus laevis frogs using an aseptic technique. Xenopus laevis were kept in tanks – usually 6–8 animals per tank – and were exposed to a 12/12 hours dark/light cycle. All Xenopus laevis surgeries were performed under general anesthesia, obtained by immersion in a 0.2% solution of tricaine methane sulfonate (MS-222) adjusted to pH 7.4 for 15–20 min. Depth of anesthesia was assessed by loss of the righting reflex and loss of withdrawal reflex to a toe pinch. After surgery animals were singly housed for 48 h. Frogs were monitored daily for 1 week post-operatively to ensure the absence of any surgery-related stress. Post-operative analgesics were not routinely used. Considering the simplicity of the procedure, the lack of complications, the effectiveness of anesthetic regimens and reductions in the number of animals likely to occur compared to the number that would be required if only one surgery were permitted, multiple surgeries on a single animal were performed. Individual donors were used up to five times, conditional upon the relative health of an individual animal. Recovery time between oocyte collections from the same animal was maximized by rotation of the frogs being used. A minimum recovery period of 1 month was ensured between ovarian lobe resection from the same animal to avoid distress. Evidence of surgery-related stress resulted in an extended rest period based on recommendations from the veterinary staff. After the fifth terminal surgery frogs were humanely sacrificed through anesthesia overdose via 2 h of immersion in a 5 g/l MS-222 solution adjusted to pH 7.4.

### Data availability statement

The datasets generated during and/or analysed during the current study are available from the corresponding author on reasonable request.

## Electronic supplementary material

Supplementary Information
