# Supplementary material for: New views on phototransduction from atomic force microscopy and single molecule force spectroscopy on native rods
Source: Sci Rep. 2017 Sep 20;7:12000. doi: 10.1038/s41598-017-11912-8 (PMC5607320; doi:10.1038/s41598-017-11912-8)
Supplement: Supplementary file 1 — Supplementary Information [file 41598_2017_11912_MOESM1_ESM.pdf]

Supplementary Information

New views on phototransduction from atomic force microscopy and single molecule force spectroscopy on native rods.

Sourav Maity, Nina Ilieva, Alessandro Laio, Vincent Torre* and Monica Mazzolini*

International School for Advanced Studies (SISSA-ISAS) via Bonomea 265, 34136 Trieste (Italy)
*corresponding authors (mazzolin@sissa.it - torre@sissa.it)

SUPPLEMENTARY INFORMATION 1

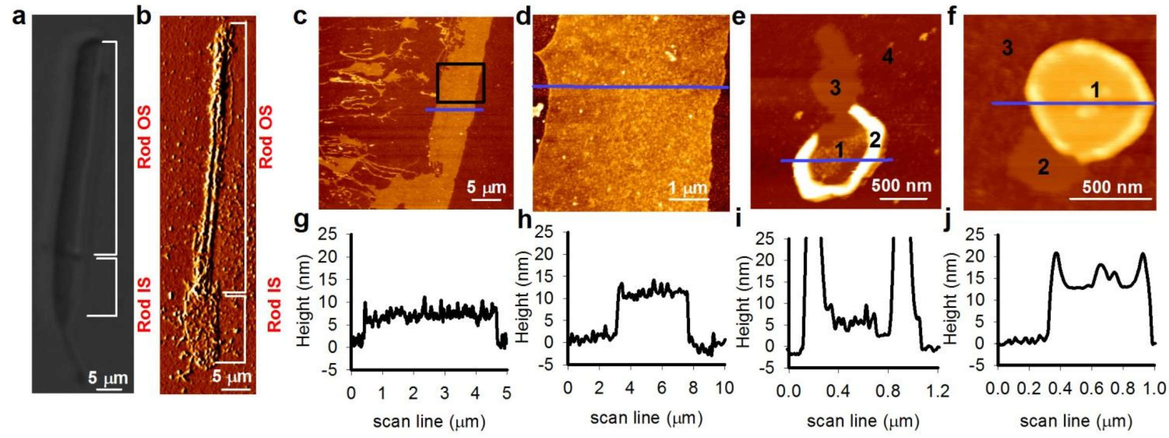

Supplementary Figure 1: Sample preparation. a: Example of an IR light image of an intact rod cell from *Xenopus laevis* frog. **b:** representative AFM scanning image of the cytoplasmic site of rod cell plasma membrane after absorption on mica surface in the presence of recording solution. **c:** AFM topography of a native rod OS plasma membrane patch. **d:** enlargement of the AFM image of plasma membrane patch underlined in the black box in panel **c. e:** AFM topography of an open, spread-flattened native disc; four different surface types are evident: the centre of the disc with a height profile around 6 nm with densely packed rhodopsin (1); the disc rim region (2); co-isolated lipid surface without any protein (3) and mica surface (4). **f:** AFM topography of an intact rod OS disc membrane; three different surface types are evident: the centre of the disc with a height profile around 14 nm with densely packed rhodopsin (1); co-isolated lipid surface without any protein (2) and mica surface (3). **g-h**: height profile taken along the blue rod OS section in panel **c** and **d** respectively; the height of the plasma membrane was around 8±1.8 nm (n=22). **i-j:** height profile taken along the blue rod OS section; discs with one or two lipid bilayers had a height of 6.5±1.8 (n=25) and 14±1.7 nm (n=18) in panel **e and f,** respectively.

SUPPLEMENTARY INFORMATION 2

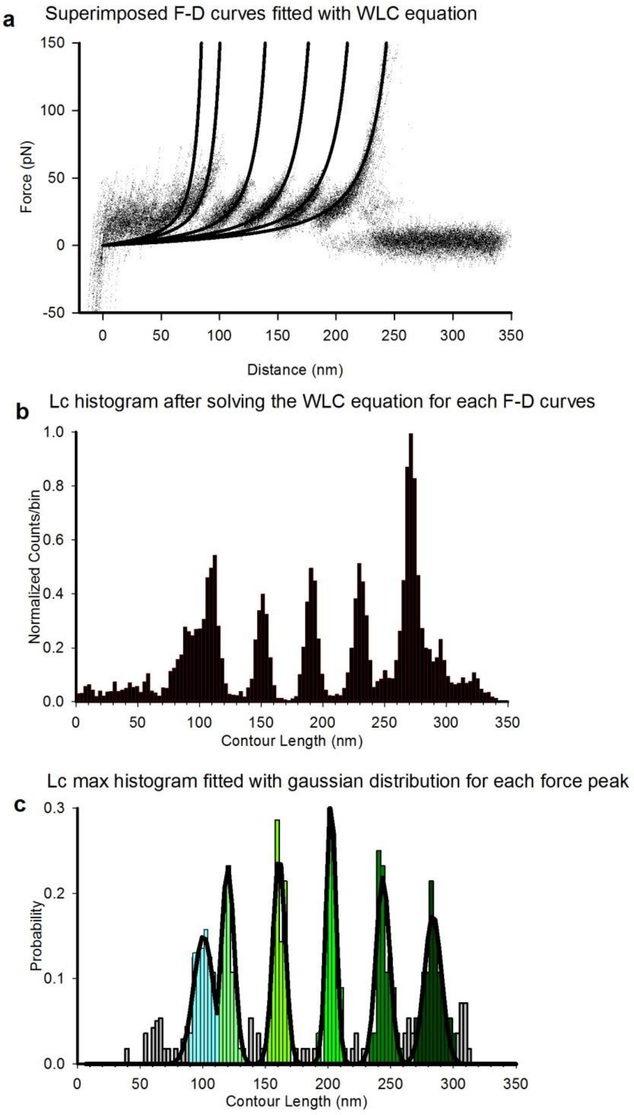

Supplementary Figure 2: Procedure for the determination of the probability of force peaks from the Lc histogram: Step one provided the superposition of all the F-D curves obtained from the clusterization. **a:** example of a superposition of 67 F-D curves from CNGA1 unfolding, continuous line provided a representative WLC fittings to each force peak. Step two is solving the WLC equation for each curve at each point provided the global Lc histogram. **b:** global Lc histogram obtained from solving the WLC equation for each point of all the 67 F-D curves from panel a. Step three is to obtain the Lc maximum histogram, and fitting with Gaussian function for each distribution in Lc histogram provided the probability for each force peak. **c:** representation of a Lc maximum histogram obtained from panel b, the continuous lines represent the Gaussian fitting for individual distribution in Lc histogram.

SUPPLEMENTARY INFORMATION 3

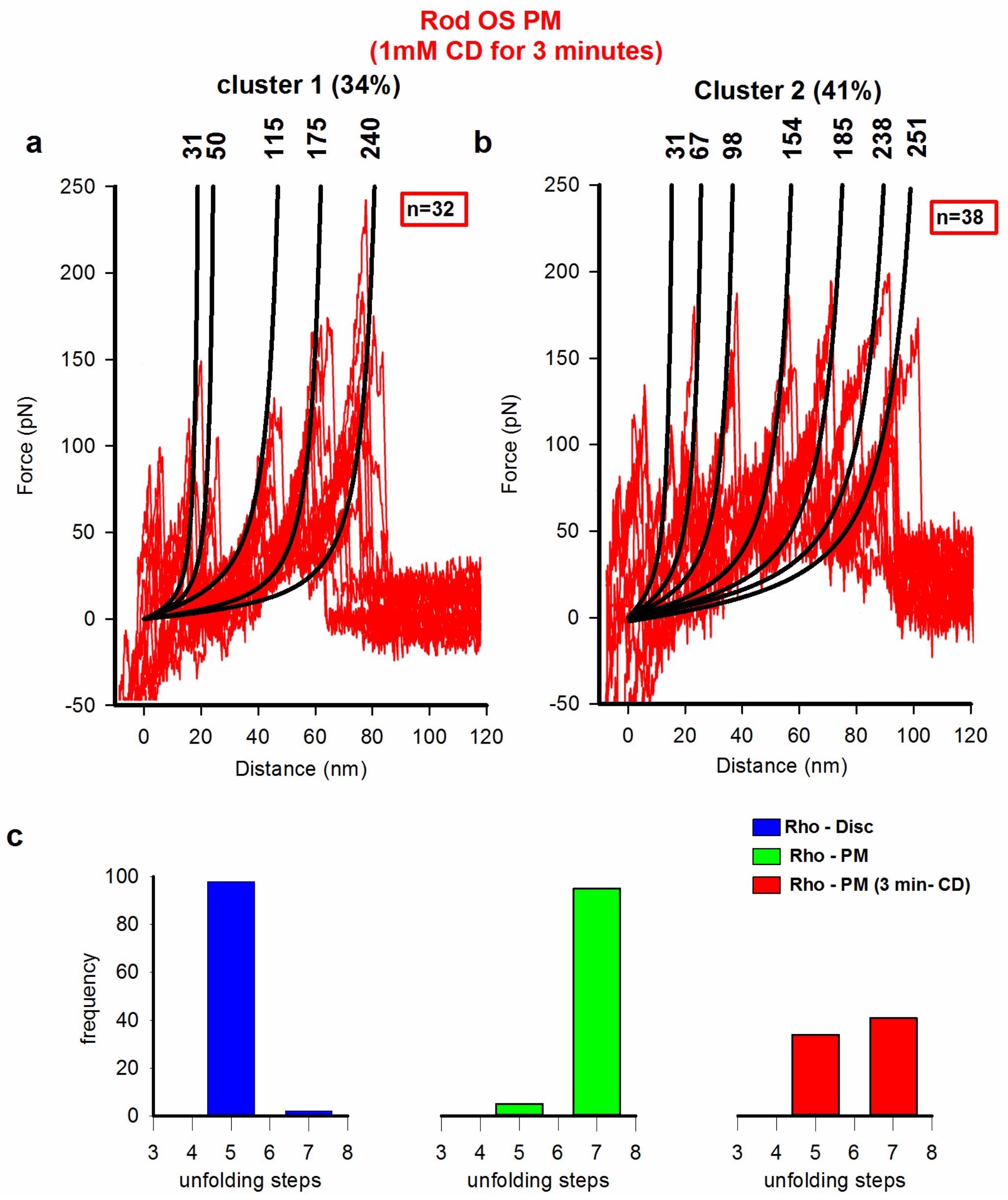

Supplementary Figure 3. Unfolding of rhodopsin from the OS plasma membrane treated with cyclodextrin. a: superposition of 4 F-D curves for Cluster 1 after the application of 1 mM cyclodexstrin (here named CD) for 3 minutes; the continuous lines represent the fitting of WLC model to each force peak. The percentage represents the probability of occurrence. **b:** as in panel a but with superposition of 4 F-D curves for Cluster 2. **c:** Plots representing the frequency versus the number of unfolding steps of rhodopsin in the different membranes with and without cyclodextrin.

SUPPLEMENTARY INFORMATION 4

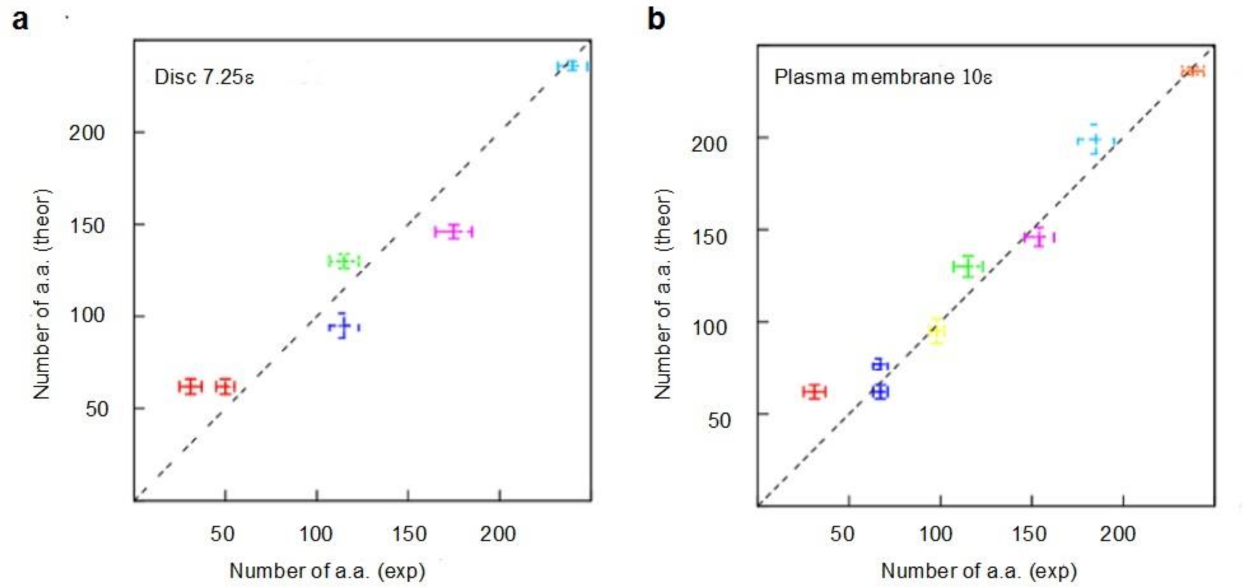

Supplementary Figure 4. a, **b**: correlation between the experimental values of n, the number of unfolded amino acids, as deduced by the values of L_c and the theoretical values of n, obtained as described in figure 6 (main text). The panel **a** corresponds to the simulation with ε_{MEMBR}=7.25ε, panel **g (fig.6)**, compared with the experimental values for the plasma membrane (Fig. 4b). The panel **b** corresponds to the simulation with ε_{MEMBR}=10ε, panel **e (fig. 6)**, compared with the experimental values for the discs (Fig. 4c). The colour map is the same as the one used in the top panels. The points with the same colouring (two blue points in panel **a** and two red points in panel **b**) correspond to ambiguous cases, in which, for instance, a single experimental peak may be associated with two theoretical peaks (and vice versa).

SUPPLEMENTARY INFO 5

Validation of the membrane potential.

In order to further validate the capability of the membrane potential V^{MEMBR} to capture the hydrophobic effect of the membrane we performed four additional simulations with our model using the same set of MD parameters, $\varepsilon_{MEMBR}=10\varepsilon$ and $v_{PULL}=0$ (no pulling force). For each simulation the number of hydrophobic residues in the initial configuration was artificially changed. We considered the amino acid LEU, which is the most abundant transmembrane hydrophobic residue in rhodopsin, and varied the number of times it has been assigned as hydrophobic in our model (1, 4 , 11 or 23 times less; 23 is the total number of transmembrane LEUs in rhodopsin). For each configuration we computed the average potential energy from the MD run and the SASA occupied by hydrophobic residues (A_{HPHOB}). In Supplementary Figure 5, the energy is plotted as a function of A_{HPHOB}. As A_{HPHOB} is increasing the energy is decreasing due to the hydrophobicity effects included in the membrane potential, V_{MEMBR}. This indicates that the functional form we use is able to capture, at least qualitatively, the effect of transferring a hydrophobic moiety into an hydrophobic environment: the larger the area of the moiety, the lower the average energy.

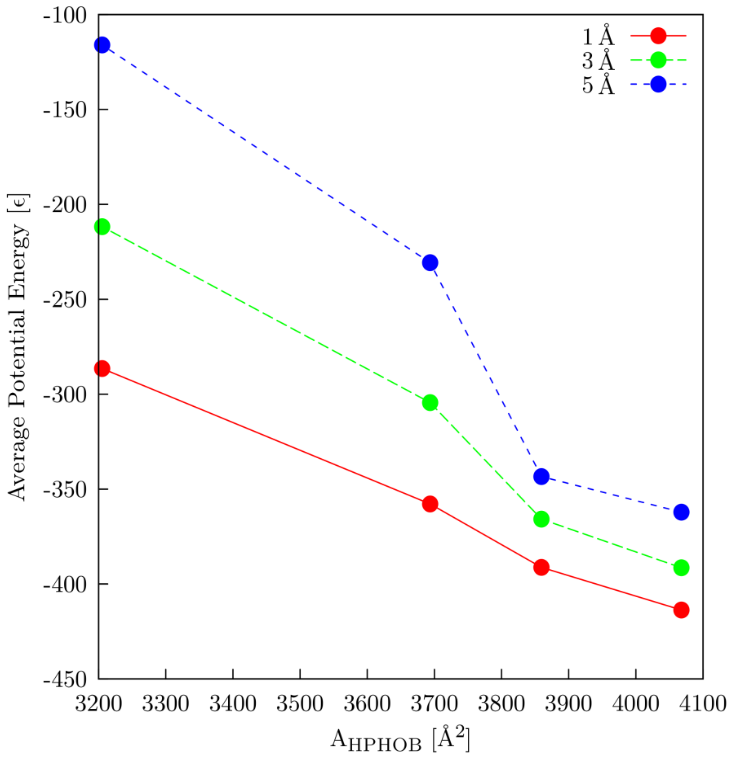

Supplementary Figure 5. Plot of energy as a function of A_{HPHOB} at different thickness of lipid intermediates.

In a typical lipid bilayer the intermediate region connecting the fully hydrated polar headgroups to the fully dehydrated hydrophobic tails has a thickness of approximately 3 Å [1]. This is the rationale behind the choice of this parameter in the V^{MEMBR} formula . However, we also verified that the exact choice of this parameter does not affect the qualitative behaviour of V^{MEMBR}. We

performed the same set of 4 MD simulations described above for cutoff 1 and 5 Å. In the figure, we show that the trend of the average energy as a function of the hydrophobic area remains qualitatively similar even if the thickness parameter is changed rather dramatically.

The setup of the all-atom molecular dynamics simulation.

We carried out molecular dynamics with the software Gromacs 4.6.7 [2]. Rhodopsin (PDB code: 1U19) is oriented following the OPM [3] database model and embedded in a pre-equilibrated lipid bilayer with 128 DPPC and 3655 water molecules [4] using the g_membed [5] tool of Gromacs. The protein charge, -3, is neutralized with 3 sodium (Na+) ions. The box is enlarged in the z-direction to yield the dimensions 6.88x6.91x17.33 nm, resulting in a system of 1 protein, 118 DPPC and 20826 water molecules and 3 Na+. Periodic boundary conditions are applied. The force field GROMOS96 53a6 [6] with included Berger lipids parameters [7] for the DPPC molecules is used. All bonds are constrained to their equilibrium lengths with the LINCS algorithm [8]. The non-bonded interactions are described by Lennard–Jones potential with a cutoff at 1.2 nm. The electrostatic interactions are estimated by the particle mesh Ewald (PME) method [9] with cutoff of 1.2 nm. The equations of motion are integrated with the leap-frog algorithm, using a time step of 2 fs. The equilibration is performed in three steps: (1) The system was first heated to a temperature of 323 K in 1 ns keeping the protein backbone fixed; (2) then a 7 ns run in a NPγT ensemble is performed, with a surface tension equal to 28 mN/m (corresponding to a -55.56 bar pressure) in the x and y directions and 1 bar in the z direction with a semi isotropic Parrinello-Rahman barostat [10]; temperature is kept fixed at 323 K with Berendsen thermostat [11]; (3) Finally, a 70 ns production run is performed.

Estimating the effect of membrane hydrophobicity on the flexibility of rhodopsin.

A thermodynamic measure of the effect of a change in the cholesterol content in the membrane is given by the partition coefficient per unit area of a hydrophobic molecule between a cholesterol-free membrane and a membrane with a given cholesterol content. This quantity can be in principle measured in an experiment, and it has been estimated for a few compounds by atomistic simulations (see below). Denoting this partition coefficient by γ, we model the effect of the change in hydrophobicity of the membrane by adding to the ordinary potential energy function an extra term of the form

$$V_\gamma(x) = -\gamma A(x) \qquad (S1)$$

where A(x) is the SASA in configuration x This term vanishes if the partition coefficient is zero, otherwise it favors configurations with large A. The functional form we have chosen is consistent with the mesoscopic definition of the hydration free energy, which should be proportional to the surface of the molecule. The same assumption is done in several implicit solvation models [12],[13].We then estimate the change in the probability distribution as a function of a geometric observable, for example the angle α, due to the extra term. The joint probability distribution as a function of α and of the SASA A is given, for γ=0, by

$$P_0(\alpha, A) = \int dx\delta(\alpha(x) - \alpha)\delta(A(x) - A)\, P(x) \qquad (S2)$$

where P(x) is the canonical probability distribution of the system. If $\gamma \neq 0$ the probability distribution is

$$P_\gamma(\alpha, A) = \int dx\delta(\alpha(x) - \alpha)\delta(A(x) - A)\, P(x) e^{\frac{\gamma \mathrm{A(x)}}{k_B T}} = c e^{\frac{\gamma A}{k_B T}} P_0(\alpha, A) \qquad (S3)$$

where c is a normalization constant. This equation allows estimating the probability distribution for any value of γ from the probability distribution measured in a reference condition, for example in a cholesterol-free membrane. Finally, the probability distribution as a function of α alone is given by

$$P_\gamma(\alpha) = \int dA P_\gamma(\alpha, A) = c \int dA\, e^{\frac{\gamma A}{k_B T}} P_0(\alpha, A) \qquad (S4)$$

The probability distribution $P_0(\alpha, A)$ entering in this equation is estimated from the molecular dynamics trajectory on a 100x100 regular grid ranging between 2.58 and 2.83 in α, between 4009.88 and 4737.92 in A_{HPHOB} using a Gausian kernel estimator [14] with Gaussian variance equal to 4 grid spacing in both directions. The protein residues considered as hydrophobic transmembrane are listed in Table 1.

Based on the same model, we estimate the change in the relative population of the metarhodopsin and rhodopsin as a function of the cholesterol concentration. We here provide only a rough estimate, based on the value of the SASA measured in the crystal structures of metarhodopsin II (3PXO) and rhodopsin (1U19). Denoting by $\frac{P^{METARHOD}(c)}{P^{RHOD}(c)}$ the ratio between the population of metarhodopsin and rhodopsin in a membrane characterized by a concentration c of cholesterol, we have

$$\frac{P^{METARHOD}(c)}{P^{RHOD}(c)} = exp\left[\frac{-\gamma(c)}{k_B T}(A_{HPHOB}^{METARHOD} - A_{HPHOB}^{RHOD})\right]\frac{P^{METARHOD}(0)}{P^{RHOD}(0)} \qquad (S5)$$

where $A_{HPHOB}^{METARHOD}$ and A_{HPHOB}^{RHOD} are the SASA of the hydrophobic residues in the two conformations and $\gamma(c)$ is the partition coefficient per unit area of a hydrophobic molecule between a pure membrane and a membrane with a cholesterol concentration c. $\gamma(c)$ is estimated from ref [15] using the value of triethylamine (TEA) in membranes composed of DOPC with cholesterol concentrations 0, 20, 30, 40 and 50 mol %. To compute the values of the partition coefficient we used the relations:

$$\mathrm{P}^{\mathrm{TEA}}(c) = exp\left[\frac{A^{TEA}}{k_B T}\gamma(\mathrm{c})\right]\mathrm{P}^{\mathrm{TEA}}(0) \qquad (S6)$$

$$\gamma(c) = \frac{\left(\ln\mathrm{P}^{\mathrm{TEA}}(c) - \ln\mathrm{P}^{\mathrm{TEA}}(0)\right)}{A^{TEA}} k_B T \qquad (S7)$$

where $\mathrm{P}^{\mathrm{TEA}}(c)$ corresponds to the probability distribution for TEA as a function of the cholesterol concentration, c (values extracted from Fig.4B, ref [15]), $\gamma(c)$ is the partition coefficient between the two lipid environments and A^{TEA} the SASA of a single triethylamine molecule. The values for γ are presented in Table 2. In this definition γ estimates the surface tension per unit area of a hydrophobic moiety. The results are largely insensitive to the choice of the molecule used to compute the surface tension per unit area, if this is hydrophobic. For example, for pyridine in membranes composed of DOPC with cholesterol concentration 40 mol % we have $\frac{\gamma^{PYR}}{A^{PYR}} = 0.0018$, a value similar to the one observed for TEA $\frac{\gamma^{TEA}}{A^{TEA}} = 0.0014$ (values extracted from Fig. 6C and D, ref. [15]).

able 1: Hydrophobic transmembrane residues and their numbers in rhodopsin (PDB:1U19).

**HYDROPHOBIC RESIDUES**	**Number**
ILE	48, 54, 75, 123, 133, 154, 205, 213, 214, 217, 219, 255, 256, 259, 263, 275, 286, 290, 305, 307
LEU	40, 46, 47, 50, 57, 59, 72, 76, 77, 79, 84, 95, 99, 112, 119, 125, 128, 131, 165, 172, 216, 262, 266

VAL	81, 87, 129, 130, 157, 162, 173, 204, 209, 210, 218, 254, 258, 271, 300, 304
PHE	37, 45, 52, 56, 85, 88, 91, 115, 116, 159, 203, 208, 212, 220, 221, 261, 273, 276, 287, 293, 294
ALA	41, 42, 80, 82, 117, 124, 132, 153, 158, 164, 166, 168, 169, 260, 269, 272, 292, 295, 299

Table 2: Partition coefficients values for triethylamine (TEA) estimated at different cholesterol concentrations in DOPC lipid membrane with cholesterol concentration 0 mol % as reference value.

**Cholesterol concentration**, c [mol %]	**Partition coefficient**, γ, $[kcal\ mol^{-1}\ Å^{-2}]$
0	0
20	$7.061 \cdot 10^{-4}$
30	$2.889 \cdot 10^{-4}$
40	$1.027 \cdot 10^{-3}$
50	$8.987 \cdot 10^{-3}$

Ethics Statement: All studies were approved by the SISSA's Ethics Committee according to the Italian and European guidelines for animal care (d.l. 116/92; 86/609/C.E.). Xenopus leaves frogs were humanely killed through anaesthesia overdose via 2 h of immersion in a 5 g/l solution of tricaine methane sulfonate (MS-222) adjusted to pH 7.4.

REFERENCES SUPPLEMENTATY

[1] Tristram-Nagle, S., Nagle, J., Structure of lipid bilayers. *BiochimicaetBiophysicaActa*, 1331, 235-270, (1997)
[2] van der Spoel D,Lindahl E, Hess B, and the GROMACS development team (2014) GROMACS User Manual version 4.6.7, *www.gromacs.org*
[3] Lomize MA, Lomize AL, Pogozheva ID, Mosberg HI (2006) OPM: Orientations of Proteins in Membranes database. *Bioinformatics***22**, 623-625.
[4] Tieleman DP, Berendsen HJC (1996) Molecular dynamics simulations of fully hydrated DPPC with different macroscopic boundary conditions and parameters. *J. Chem. Phys.***105**, 4871-4880
[5] Wolf MG, Hoefling M, Aponte-Santamaría C, Grubmüller H, Groenhof GJ (2010) g_membed: Efficient insertion of a membrane protein into an equilibrated lipid bilayer with minimal perturbation.*Comput. Chem.***31**, 2169−2174.
[6] Oostenbrink C, Villa A, Mark AE, van Gunsteren WF (2004) A biomolecular force field based on the free enthalpy of hydration and solvation: the GROMOS force-field parameter sets 53A5 and 53A6.*J. Comp. Chem.***13**, 1656-1676.
[7] Berger O, Edholm O, Jähnig F (1997) Molecular dynamics simulations of a fluid bilayer of dipalmitoylphosphatidylcholine at full hydration, constant pressure, and constant temperature. *Biophys. J.* **72**, 2002-2013.
[8] Ryckaert JP, Ciccotti G, Berendsen HJC (1977) Numerical integration of the cartesian equations of motion of a system with constraints: molecular dyanmics of n-alkanes.*J. Comput. Phys.***23**, 327–341.
[9] Darden T, York D, Pedersen L (1993) Particle mesh Ewald: An N.log(N) method for Ewald sums in large systems.*J. Chem. Phys.***98**, 10089–10093.

[10] Parrinello M, Rahman A (1981) Polymorphic transitions in single crystals: A new molecular dynamics method.*J. Appl. Phys.***52**, 7182−7190.
[11] Berendsen HJC, Postma JPM, DiNola A, Haak JR (1984) Molecular dynamics with coupling to an external bath.*J. Chem. Phys.***81**, 3684–3691.
[12] Simonson, T., Roux, B., Implicit solvent models.*Biophysical Chemistry,* 78, 1-20 (1999)
[13]Brooks III,C., Feig, M., Im, W.,An implicit membrane generalized Born theory for the study of structure, stability, and interactions of membrane proteins.*Biophys. J.* 85, 2900-2918 (2003)
[14] Parzen E (1962) On estimation of a probability denstiy function and mode. *Ann. Math. Statist.***33(3)**, 1065-1076.\
[15] Zocher F, van der Spoel D, Pohl P, Hub JS (2013) Local partition coefficients govern solute permeability of cholesterol-containing membranes. *Biophys J*. **105**, 2760-2770.
